# Elevated Plasma *α*-Defensins (HNP1–3) Levels Correlated with IgA1 Glycosylation and Susceptibility to IgA Nephropathy

**DOI:** 10.1155/2016/8123138

**Published:** 2016-08-02

**Authors:** Yuan-yuan Qi, Xu-jie Zhou, Fa-juan Cheng, Hong Zhang

**Affiliations:** ^1^Renal Division, Peking University First Hospital, Beijing 100034, China; ^2^Peking University Institute of Nephrology, Beijing 100034, China; ^3^Key Laboratory of Renal Disease, Ministry of Health of China, Beijing 100034, China; ^4^Key Laboratory of Chronic Kidney Disease Prevention and Treatment, Peking University, Ministry of Education, Beijing 100034, China; ^5^Shandong Provincial Hospital, Shandong University, Jinan, Shandong 250021, China

## Abstract

*Aim*. IgA nephropathy (IgAN) is the most common form of glomerulonephritis. Recent genome-wide association study (GWAS) suggested that DEFA locus (which encodes *α*-defensins) may play a key role in IgAN.* Methods*. The levels of *α*-defensins in 169 IgAN patients and 83 healthy controls were tested by ELISA.* Results*. We observed that *α*-defensins human neutrophil peptides 1–3 (HNP1–3) in IgAN patients were elevated compared with healthy controls. The mean levels of *α*-defensins of 83 healthy controls and 169 IgAN patients were 50 ng/mL and 78.42 ng/mL. When the results were adjusted to the mean levels of *α*-defensins of IgAN patients, the percentage of individuals with high levels of *α*-defensins increased in IgAN patients (22.5%) compared to healthy controls (9.6%) (*p* = 0.013). The elevation of *α*-defensins in IgAN patients was independent of renal function or neutrophil count, which were major sources of *α*-defensins in circulation. More importantly, negative correlation was observed between galactose-deficient IgA1and *α*-defensins.* Conclusion*. As *α*-defensin is a lectin-like peptide, we speculated that it might be involved in IgA galactose deficiency. The data implied that patients with IgAN had higher plasma *α*-defensins levels and high *α*-defensins correlated with IgA galactose deficiency, further suggesting a pathogenic role of *α*-defensins in IgAN.

## 1. Introduction

IgAN is the most common cause of end-stage renal disease (ESRD) among Asian populations [1]. The diagnostic hallmark of IgAN is the predominance of deposition with immune complex containing IgA in mesangial regions, but the pathogenetic factors remain obscure. Multiple studies have established that aberrantly glycosylated IgA1 plays an important role in the pathogenesis of IgAN [2–6]. In patients with IgAN, some circulating IgA1 are deficient of O-glycans. Furthermore, mesangial immune deposits eluted directly from glomeruli of IgAN patients contained Gd-IgA1 [7, 8]. Our previous study discovered that the elevation of serum galactose-deficient IgA1 is associated with a poor prognosis in IgAN [9]. On the basis of the potential pathogenic role of Gd-IgA1 in the development of IgAN, the mechanism of the glomerular mesangium deposition of IgA1 immune complexes remained to be an import issue. Recent advances implicated that genetic factor, especially in promoting the overproduction of an aberrant form of IgA1, plays a key role in IgAN. More recently, several genome-wide association studies and candidate gene studies confirmed that single nucleotide polymorphisms (SNPs) in* DEFA* locus were strongly associated with IgAN susceptibility, further supporting the likely key role of mucosal immunity in IgAN [10–14].

HNP1–3, encoded by* DEFA1* and* DEFA3*, are part of the *α*-defensins family of peptides, which in humans include six known members and are almost identical in sequences. They are normally synthesized in neutrophil precursor cells and released by mature circulating neutrophils at inflammatory sites [15]. *α*-defensins are generally considered to be direct effectors of innate antimicrobial immunity to kill and/or inactivate a particular spectrum of bacteria, fungi, or some enveloped viruses in vitro [15]. In addition, *α*-defensins might also be a component of the effector of adaptive immunity mediating B- and T-cell interactions linking innate and adaptive immunity [16].

In certain disease states, plasma or local *α*-defensins would depart from normal levels. *α*-defensins levels were elevated in plasma of SLE patients and could serve as a marker for disease activity [17, 18]. In patients with diabetic nephropathy, HNP1–3 concentrations are increased and there was an independent relationship between estimated GFR (Cockcroft–Gault) and HNP1–3 [19]. Serological studies of defensins would provide crucial insight in exploring the pathogenesis and the development of diseases. As for IgA nephropathy, the current progress is in genetic field along with multiple SNP identified contributed to the IgAN susceptibility. Since serological investigation is absent in IgAN, we conducted a case-control study to check the plasma levels of *α*-defensins in IgAN patients.

## 2. Materials and Methods

### 2.1. Subjects

In the present study, 169 IgAN patients, whose plasma were available and were followed up at least 12 months, were enrolled. The diagnosis of IgAN was proved by renal biopsy. 83 healthy controls were blood donors with matched gender and age with the IgAN patients. Patients with IgAN were confirmed by renal biopsy and those with secondary IgAN or other comorbid renal diseases such as systemic lupus erythematosus, vasculitis, or Henoch-Schonlein purpura were not included. Clinical data, including age (years), gender (male/female, %), systolic blood pressure (SBP) (mmHg), diastolic blood pressure (DBP) (mmHg), neutrophil count (*∗*10^9^/L), prodromal infection (%), elevated C-reactive protein (CRP) levels (%), erythrocyte sedimentation rate (ESR) (mm/1st h), triglycerides (TG) (mmol/L), total cholesterol (TCHOL) (mmol/L), high-density lipoprotein cholesterol (HDL) (mmol/L), low-density lipoprotein cholesterol (LDL) (mmol/L), hyperlipidemia (%), uric acid (*μ*mol/L), serum creatinine (Scr) (*μ*mol/L), estimate glomerular filtration rate (eGFR) (mL/min per 1.73 m^2^), stage 1, 2, 3, 4, and 5 CKD (%), galactose-deficient IgA1 (Gd-IgA1) (U/mL, median, IQR), total IgA (*μ*g/mL, median, IQR), plasma IgA1 (*μ*g/mL, median, IQR), initial proteinuria (g/day), urinary red blood cell (RBC) count (/*μ*L), gross hematuria (%), 24-hour total urine protein (UTP) (g/day), histological grading, I, II, III, IV, and V (%), Oxford classification M (%), E (%), S (%), and T (%), angiotensin-converting enzyme (ACE) inhibitors or angiotensin II receptor blockers (ARBs) (%), and prednisone (%), were collected at the time of renal biopsy. The composite end point was defined as 50% decline from baseline eGFR or end-stage renal disease (ESRD) or death. For the purpose of this study, ESRD was defined as eGFR < 15 mL/min per 1.73 m^2^ or need for renal replacement therapy (such as hemodialysis, peritoneal dialysis, or renal transplantation). The modification of diet in renal disease formula was used to calculate the eGFR [20]. At least two independent pathologists graded and reviewed the renal biopsy specimens and reached a consensus. The histological lesions were classified according to HASS grading (I–V) from mild lesions to severe lesions [21]. Hyperlipidemia was assigned when any of the TG, TCHOL, and LDL indexes was above the normal ranges of the department of laboratory of our hospital. This study was approved by the medical ethics committee of Peking University First Hospital and written informed consent was obtained from all the patients.

### 2.2. Detection of Plasma HNP1–3, IgA, Plasma IgA1, and Gd-IgA1 by ELISA

Plasma IgA, IgA1, and Gd-IgA1 levels were detected by ELISA as previously published [9]. Briefly for Gd-IgA1, 2.5 mg/mL F(ab′)_2_ fragment of goat anti-human IgA (Jackson ImmunoResearch Labs, West Grove, PA) was incubated in high-binding MaxiSorp 96-well plates (Nalge-Nunc, Rochester, NY) and blocked with 1% bovine serum albumin (Sigma Chemical Company, St Louis, MO) in PBS 0.05% Tween 20. The standard curve was made by duplicates of twofold dilutions of serum samples with a range from 1 : 2000 to 1 : 16,000 for Gd-IgA1. To quantitate Gd-IgA1, a standard consisting of a polymeric Gd-IgA1 protein isolated from a patient with multiple myeloma was used and the terminal sialic acid from O-linked N-acetylgalactosamine on bound samples and the standard IgA1 myeloma protein were removed by incubation with 1 mU/well neuraminidase (Roche Diagnostic, Indianapolis, IN) in 0.01 mol/L acetate buffer pH 5 for 3 h at 37°C. Then they were incubated with 100 mL biotin-labeled HAA (1 : 500 dilution, Sigma) for 3 h at 37°C. After washing, they were incubated with 100 mL mouse anti-human monoclonal antibody (1 : 50,000 dilution, Sigma) for 1 h at 37°C followed by an incubation with horseradish peroxidase-ExtrAvidin (Sigma) for 1 h at 37°C. 1 mol/L sulfuric acid was used to stop the color reaction and the absorbance was measured at 490 nm with an EL312 Bio-Kinetics microplate reader (Bio-Tek Instruments, Winooski, VT). The determination of Gd-IgA1 concentration in the tested samples was calculated with the DeltaSoft II program (BioMetallics, Princeton, NJ) by interpolating the optical densities on calibration curves, constructed using a Gd-IgA1 myeloma protein. Influenced by galactose in the standard IgA1 myeloma protein, the concentration of Gd-IgA1 was expressed in U/mL. The HAA-ELISA was repeated with 108 randomly selected patients' samples for intra-assay variability.

Plasma *α*-defensin concentration of IgAN patients was quantified by ELISA using a commercial human HNP1–3 ELISA Kit (Hycult Biotechnology B.V., Uden, Netherlands) with the measurable concentration ranges from 156 to 10,000 pg/mL. Briefly, plasma samples were stored at −80°C after being separated by centrifugation at 1500 rpm for 15 min at room temperature and clarification centrifugation at 3000 rpm. According to manufacturer's instructions, samples and standards are incubated for 60 min in microtiter wells coated with human HNP1–3 antibodies. After 4 times of thorough washing, the second antibody containing biotinylated tracer antibody was added to capture human HNP1–3 for 60 min and then washed again and incubated with treptavidin-peroxidase conjugate for 60 min. The reaction was stopped with 1 mol/L sulfuric acid and the absence was measured at 450 nm with an EL312 Bio-Kinetics microplate reader (Bio-Tek Instruments, Winooski, VT). To assess the degree of intra-assay variability, one sample had been repeated 3 times to minimize the deviation.

### 2.3. Statistical Analyses

Means and standard deviations were used to summarize statistics for normally distributed quantitative variables. We used median and IQR to summarize nonnormally distributed variables and ratios and percentages for categorical data. Student's* t*-test (two groups) or analysis of variance (multiple groups) was used to compare differences in means for continuous variables. Differences in proportions were tested by *χ* ^2^ test. The association of plasma HNP1–3 levels and the primary outcome were analyzed by Cox proportional hazards models. We used natural log to transform HNP1–3 and Gd-IgA1 as it was highly skewed to the right in this group of patients. Statistical analysis was achieved with the SPSS 16.0 software package (SPSS Inc., Chicago, IL). A two-tailed *p* value < 0.05 was considered statistically significant.

## 3. Results

### 3.1. Patient Baseline Characteristics and Clinical Outcome

As is shown in Table 1, there were 86 (50.9%) males and 83 (49.1%) females with mean age at the time of kidney biopsy of 32.7 ± 10.5 years. The median proteinuria of IgAN patients on biopsy was 1.98 ± 1.90 g per 24 h (range: 0.14–13.72 g/24 h) and median Scr was 91 *μ*mol/L (range: 54–789 *μ*mol/L). The average eGFR was 81.66 ± 26.84 mL/min per 1.73 m^2^ (range 6.30–164.90 mL/min per 1.73 m^2^). Systolic blood pressure was 124 ± 17 mmHg and diastolic blood pressure was 80 ± 12 mmHg, with 49 patients (29%) being hypertensive at baseline. The distribution by Haas grades I, II, III, IV, and V was 10.1%, 0%, 34.3%, 42%, and 13.6%, respectively. The median follow-up time was 48 months (range: 12–96 months, Table 1). 165 (97.6%) patients received ACE inhibitors or ARBs therapy and 79 (46.7%) received oral corticosteroids alone or combined with other immunosuppressive agents during the follow-up period. In total, 30 patients reached the composite end point of 50% decline in eGFR, ESRD, or death.

### 3.2. Levels of HNP1–3 in IgAN Patients

**Figure 1:**
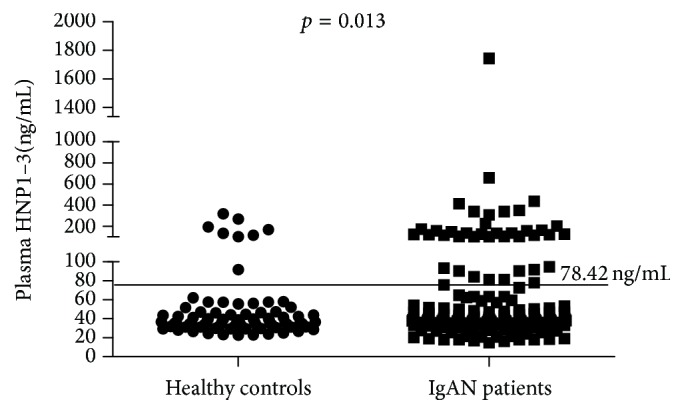
Plasma *α*-defensins (HNP1–3) levels of IgAN patients and healthy controls. The line was mean levels of *α*-defensins for 169 IgAN patients (78.42 ng/mL) defined as the cut-off for dividing patients into two groups with high (above 78.42 ng/mL) and low (below 78.42 ng/mL) *α*-defensins levels.

We first set out to determine the levels of the HNP1–3 in the plasma of our IgAN patients by ELISA. The mean levels of plasma *α*-defensins 83 healthy controls and 169 patients with IgA nephropathy were 50 ng/mL and 78.42 ng/mL. As can be shown in Figure 1, some patients with IgAN showed marked value of *α*-defensin. When the results were adjusted to the value of the mean level of *α*-defensin in IgAN patients, it was shown that the percentage of individuals with high levels of *α*-defensins increased in patients (22.5%) comparing with that in healthy controls (9.6%) (*p* = 0.013, OR 2.719, 95% CI 1.206–6.135).

### 3.3. The Levels of HNP1–3 Correlation with IgAN Clinical Characteristics and Prognosis

**Figure 2:**
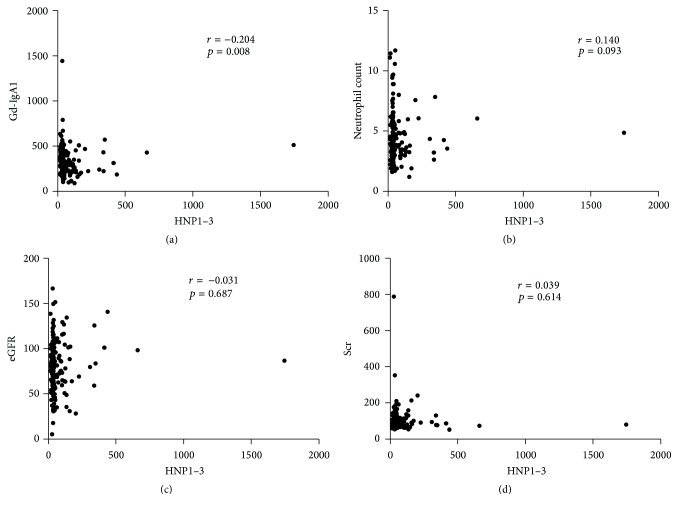
Two-tailed bivariate correlations between plasma levels of HNP1–3 (ng/mL) and IgAN clinical ((a) Gd-IgA1, U/mL; (b) neutrophil count, *∗*10^9^/L; (c) eGFR, mL/min per 1.73 m^2^; (d) Scr, *μ*mol/L) characteristics.

To test the clinical manifestations differences between patients with high and low *α*-defensins levels, the median *α*-defensins level of healthy controls (78.42 ng/mL) was defined as the cut-off for dividing patients into two groups. Low plasma *α*-defensins levels were defined as levels below 78.42 ng/mL. High plasma *α*-defensins levels were defined as levels above 78.42 ng/mL. 131 (77.5%) IgAN patients were assigned to the group with low (below 78.42 ng/mL) *α*-defensins levels and 38 (22.5%) IgAN patients were assigned to the group with high (above 78.42 ng/mL) *α*-defensins levels. The main clinical manifestations demonstrated that there were no differences in age, sex, blood pressure, initial proteinuria, and eGFR, in two groups of patients. Serum creatinine and eGFR were not associated with the levels of *α*-defensins in IgAN patients, likely suggesting no accumulation of *α*-defensins as the result of reduced glomerular filtration rate (*r* = 0.039, *p* = 0.614 and *r* = −0.031, *p* = 0.687, resp.) (Figures 2(d) and 2(c)). In addition, there was no correlation between the neutrophil count and the levels of *α*-defensins in IgAN patients (*r* = 0.140, *p* = 0.093) (Figure 2(b)).

In the group of patients with high *α*-defensins plasma levels, Gd-IgA1 was significantly lower (median 236.98.47 U/mL and interquartile range 203.10–350.01 U/mL) than patients with low *α*-defensins levels (median 318.56 U/mL, interquartile range 242.80–405.67 U/mL, and *p* = 0.036). More importantly, we also found that low Gd-IgA1 was correlated with high *α*-defensins levels (*r* = −0.204, *p* = 0.008) (Figure 2(a)). Levels of plasma IgA or plasma IgA1 showed no significant between patients with high and low *α*-defensins (Table 1).

No association were observed in plasma HNP1–3 level with other clinical manifestations (such as SBP, DBP, prodromal infection, gross hematuria, 24-hour UTP, Scr, and eGFR) and renal outcome (Table 1).

## 4. Discussion

In this study, we measured the levels of HNP1–3 in IgAN patients. We found that the *α*-defensins in IgAN patients were elevated compared with healthy controls which was independent of renal function and neutrophil count. The high level of plasma *α*-defensins might be due to neutrophil activation instead of increased neutrophil numbers. More importantly, we also found that low Gd-IgA1 was associated with high *α*-defensins levels. On the basis of the potential pathogenic role of Gd-IgA1 in the development of IgAN, *α*-defensins may be involved in the pathogenesis of IgAN not just through serological changes.

The defensins of vertebrate animals comprised by 18–45 amino-acid residues are small cationic peptides including three subfamilies of defensins, namely, *α*-, *β*-, and *θ*-defensins. There are six human *α*-defensin peptides, namely, HNP1–3, HNP4, and HD5-6, which shows consistency in amino acids sequences to some degree. HNP1–3 and HD5 have lectin-like affinity for glycosylated molecules that can bind carbohydrates in making glycolipids, glycoproteins, and proteoglycans [22–25]. IgA1 are glycoproteins which consist of a hinge region (HR) with a high content of proline (Pro), serine (Ser), and threonine (Thr) between the first and second heavy chain constant region domains. Within the IgA1 HR, six O-linked glycan chains have been identified which have been localized to Ser/Thr residues Thr225, Thr228, Ser230, Ser232, Thr233, and Thr236 [26–30]. IgA1 O-linked glycans are composed of N-acetylgalactosamine (GalNAc) with a *β*1, 3-linked Gal. *α*2, 3-linked Gal or *α*2, 6-linked GalNAc may be capable of sialic acid attachment [31–33]. Glycosylation is the process that a carbohydrate, such as a glycosyl donor, is attached to a hydroxyl or a glycosyl acceptor. Gd-IgA1 is mainly represented as the deficiency of galactose in hinge regions which may lead to increased exposure of glycosyl in GalNAc. *α*-defensin is a lectin-like peptide, which has carbohydrate-binding activity [25]. We hypothesized that *α*-defensin could bond to exposed glycosyl in N-acetylgalactosamine of IgA1 molecular which might contribute to IgA1 aggregation and deposition in mesangial regions. Our present study supported the hypothesis showing that *α*-defensins were elevated in patients with IgAN and were negatively correlated with galactose deficiency of circulating IgA1.

In addition to their role in circulation, *α*-defensins can also play a role in local tissues. *α*-defensins can induce the secretion of inflammatory cytokines and inflammatory chemokines in lung epithelial cells promoting local inflammatory response. Besides, *α*-defensins can also facilitate the migration of fibroblasts/epithelial cell proliferation and increase extracellular matrix production [34]. Since *α*-defensins have both inflammatory and fibrosis effects in other organs, further study should be carried out in depth about their effects on renal tissue. Unfortunately, no significant difference as for M, E, S, and T classification was observed between patients with the low and high *α*-defensins levels. This negative finding might be due to certain cell lines used in the inflammatory and fibrosis effects of *α*-defensins studies without evidences from renal resident cells. Since the effect of different cell lines might not be the same, the inflammatory and fibrosis effects of renal resident cells should be further investigated. However, the correlation between the levels of *α*-defensins and Gd-IgA1 was relatively low. And no significant association of clinical manifestations was observed between patients with high and low levels of *α*-defensins. Future more widespread replications and functional assays were of special interest.

In conclusion, our study identified that the *α*-defensins in IgAN patients were elevated which was independent of renal function and neutrophil count. And we also found that high Gd-IgA1 was associated with low *α*-defensins levels. We speculate that *α*-defensins may bind Gd-IgA1 in circulation which finally deposit in the kidney promoting local inflammation and fibrosis.

## Figures and Tables

**Table 1:** Clinical and laboratory data for IgAN patients with different plasma *α*-defensins levels.

	All patients	Patients with low *α*-defensins plasma levels^1^	Patients with high *α*-defensins plasma levels^2^	*P* value
Age (years)	32.7 ± 10.5	32.5 ± 10.7	33.5 ± 9.8	0.614
Gender (male/female, %)	86 (50.9)/83 (49.1)	67 (51.1)/64 (48.9)	19 (50.0)/19 (50.0)	0.901
SBP (mmHg)	123.9 ± 16.9	124.8 ± 17.8	120.7 ± 13.3	0.184
DBP (mmHg)	79.5 ± 13.2	80.0 ± 13.8	77.8 ± 10.7	0.350
Neutrophil count (*∗*10^9^/L)	4.62 ± 2.00	4.49 ± 2.07	5.08 ± 1.71	0.147
Prodromal infection (%)	127 (75.1)/42 (24.9)	98 (74.8)/33 (25.2)	29 (76.3)/9 (23.7)	0.850
CRP (%)	112 (66.3)/7 (4.1)	82 (62.6)/5 (3.8)	30 (78.9)/2 (5.3)	0.918
ESR (mm/1st h)	18.1 ± 17.7	17.5 ± 16.9	19.8 ± 20.4	0.514
TG (mmol/L)	1.93 ± 1.51	1.98 ± 1.60	1.79 ± 1.11	0.499
TCHOL (mmol/L)	5.03 ± 1.50	4.98 ± 1.46	5.18 ± 1.663	0.457
HDL (mmol/L)	1.21 ± 0.57	1.23 ± 0.62	1.14 ± 0.33	0.384
LDL (mmol/L)	2.89 ± 0.98	2.83 ± 0.90	3.09 ± 1.19	0.153
Hyperlipidemia (%)	66 (39.1)/103 (60.9)	51 (38.9)/80 (61.1)	15 (39.5)/23 (60.5)	0.952
Uric acid (*μ*mol/L)	356.7 ± 118.3	361.5 ± 119.0	340.2 ± 115.9	0.330
Scr (*μ*mol/L)	104.8 ± 65.6	106.4 ± 71.6	99.3 ± 39.1	0.556
eGFR (mL/min per 1.73 m^2^)^3^	81.4 ± 27.8	80.9 ± 27.8	83.2 ± 28.2	0.654
Stage 1, 2, 3, 4, and 5 CKD (%)	50 (29.6), 67 (39.6), 34 (20.1), 2 (1.2)	40 (30.5), 60 (45.8), 30 (22.9), 1 (0.8)	10 (26.3), 7 (18.4), 4 (10.5), 1 (2.6)	0.224
Gd-IgA1 (U/mL, median, IQR)	303.86 (231.43–396.02)	318.56 (242.80–405.67)	236.98 (203.10–350.01)	**0.036**
Total IgA (*μ*g/mL, median, IQR)	2630.0 (2153.5–3333.5)	2658.0 (2167.0–3347.0)	2570.5 (2029.3–3365.3)	0.565
Plasma IgA1 (*μ*g/mL, median, IQR)	169.64 (138.29–207.57)	164.3 (135.3–206.4)	187.56 (148.72–216.46)	0.318
Initial proteinuria (g/day)	1.98 ± 1.90	1.89 ± 1.65	2.32 ± 2.58	0.219
Urinary RBC (/*μ*L)	286 ± 537	318 ± 599	184 ± 219	0.181
Gross hematuria (%)	129 (76.3)/40 (23.7)	99 (75.6)/32 (24.4)	30 (78.9)/8 (21.1)	0.667
24-hour UTP (g/day)	1.14 ± 1.06	1.13 ± 1.08	1.16 ± 1.02	0.886
Histological grading, I, II, III, IV, V^4^ (%)	17 (10.1), 0 (0), 58 (34.3), 71 (42.0), 23 (13.6)	10 (7.6), 0 (0), 43 (32.8), 58 (44.3), 20 (15.3)	7 (18.4), 0 (0), 15 (39.5), 13 (34.2), 3 (7.9)	0.129
Oxford classification				
M (%)	54 (32.0), 112 (66.3)	38 (29.0)/92 (70.2)	16 (42.1)/20 (52.6)	0.085
E (%)	60 (35.5), 106 (62.7)	45 (34.4)/85 (64.9)	15 (39.5)/21 (55.3)	0.436
S (%)	45 (26.6), 121 (71.6)	36 (27.5)/94 (71.8)	9 (23.7)/27 (71.1)	0.748
T (%)	109 (64.5), 36 (21.3), 21 (12.4)	86 (65.6), 28 (21.4), 16 (12.2)	23 (60.5), 8 (22.2), 5 (13.2)	0.959
*Therapy*				
ACE inhibitors or ARBs (%)	4 (2.4)/165 (97.6)	3 (2.3)/128 (97.7)	1 (2.6)/37 (97.4)	0.903
Prednisone (%)	79 (46.7)/90 (53.3)	65 (49.6)/66 (50.4)	14 (36.8)/24 (63.2)	0.165

SBP: systolic blood pressure; DBP diastolic blood pressure; CRP: C-reactive protein; ESR: erythrocyte sedimentation rate; TG: triglycerides; TCHOL: total cholesterol; HDL: high-density lipoprotein cholesterol; LDL: low-density lipoprotein cholesterol; Scr: serum creatinine; eGFR: estimate glomerular filtration rate; CKD: chronic kidney disease; 24-hour UTP: 24-hour total urine protein; Gd-IgA1: galactose-deficient IgA1; ACE: angiotensin-converting enzyme; ARB: angiotensin II receptor blocker.

^1^Low plasma *α*-defensins levels were defined as levels below 78.42 ng/mL. ^2^High plasma *α*-defensins levels were defined as levels above 78.42 ng/mL. ^3^eGFR was calculated based on MDRD formula modified population. ^4^Histological grading was classified according to pathological proposed by Haas.

## Abbreviations

IgAN: IgA nephropathy
Gd-IgA1: Galactose-deficient IgA1
GWAS: Genome-wide association study
HNP: Human neutrophil peptide
ESRD: End-stage renal disease
SNPs: Single nucleotide polymorphisms
eGFR: Estimated glomerular filtration rate
GalNAc: N-Acetylgalactosamine
ACE: Angiotensin-converting enzyme
ARB: Angiotensin II receptor blocker
CKD: Chronic kidney disease
DBP: Diastolic blood pressure
SBP: Systolic blood pressure
CRP: C-reactive protein
ESR: Erythrocyte sedimentation rate
TG: Triglycerides
TCHOL: Total cholesterol
HDL: High-density lipoprotein cholesterol
LDL: Low-density lipoprotein cholesterol
Scr: Serum creatinine
24-hour UTP: 24-hour total urine protein.

## References

[1] Levy M., Berger J. (1988). Worldwide perspective of IgA nephropathy. *American Journal of Kidney Diseases*.

[2] Amore A., Cirina P., Conti G., Brusa P., Peruzzi L., Coppo R. (2001). Glycosylation of circulating IgA in patients with IgA nephropathy modulates proliferation and apoptosis of mesangial cells. *Journal of the American Society of Nephrology*.

[3] Barratt J., Feehally J. (2005). IgA nephropathy. *Journal of the American Society of Nephrology*.

[4] Barratt J., Feehally J., Smith A. C. (2004). Pathogenesis of IgA nephropathy. *Seminars in Nephrology*.

[5] Smith A. C., de Wolff J. F., Molyneux K., Feehally J., Barratt J. (2006). O-glycosylation of serum IgD in IgA nephropathy. *Journal of the American Society of Nephrology*.

[6] Suzuki H., Moldoveanu Z., Hall S. (2008). IgA1-secreting cell lines from patients with IgA nephropathy produce aberrantly glycosylated IgA1. *Journal of Clinical Investigation*.

[7] Novak J., Julian B. A., Tomana M., Mestecky J. (2008). IgA glycosylation and IgA immune complexes in the pathogenesis of IgA nephropathy. *Seminars in Nephrology*.

[8] Allen A. C., Bailey E. M., Brenchley P. E., Buck K. S., Barratt J., Feehally J. (2001). Mesangial Iga1 in IgA nephropathy exhibits aberrant O-glycosylation: observations in three patients. *Kidney International*.

[9] Zhao N., Hou P., Lv J. (2012). The level of galactose-deficient IgA1 in the sera of patients with IgA nephropathy is associated with disease progression. *Kidney International*.

[10] Gharavi A. G., Kiryluk K., Choi M. (2011). Genome-wide association study identifies susceptibility loci for IgA nephropathy. *Nature Genetics*.

[11] Yang C., Jie W., Yanlong Y. (2012). Genome-wide association study identifies TNFSF13 as a susceptibility gene for IgA in a South Chinese population in smokers. *Immunogenetics*.

[12] Kiryluk K., Li Y., Scolari F. (2014). Discovery of new risk loci for IgA nephropathy implicates genes involved in immunity against intestinal pathogens. *Nature Genetics*.

[13] Yu X.-Q., Li M., Zhang H. (2012). A genome-wide association study in Han Chinese identifies multiple susceptibility loci for IgA nephropathy. *Nature Genetics*.

[14] Qi Y. Y., Zhou X. J., Cheng F. J. (2015). DEFA gene variants associated with IgA nephropathy in a Chinese population. *Genes & Immunity*.

[15] Lehrer R. I., Lu W. (2012). *α*-Defensins in human innate immunity. *Immunological Reviews*.

[16] Yang D., Biragyn A., Kwak L. W., Oppenheim J. J. (2002). Mammalian defensins in immunity: more than just microbicidal. *Trends in Immunology*.

[17] Sthoeger Z. M., Bezalel S., Chapnik N., Asher I., Froy O. (2009). High *α*-defensin levels in patients with systemic lupus erythematosus. *Immunology*.

[18] Cheng F.-J., Zhou X.-J., Zhao Y.-F., Zhao M.-H., Zhang H. (2015). Human neutrophil peptide 1–3, a component of the neutrophil extracellular trap, as a potential biomarker of lupus nephritis. *International Journal of Rheumatic Diseases*.

[19] Saraheimo M., Forsblom C., Pettersson-Fernholm K., Flyvbjerg A., Groop P.-H., Frystyk J. (2008). Increased levels of *α*-defensin (-1, -2 and -3) in type 1 diabetic patients with nephropathy. *Nephrology Dialysis Transplantation*.

[20] Levey A. S., Bosch J. P., Lewis J. B., Greene T., Rogers N., Roth D. (1999). A more accurate method to estimate glomerular filtration rate from serum creatinine: a new prediction equation. *Annals of Internal Medicine*.

[21] Haas M. (1997). Histologic subclassification of IgA nephropathy: a clinicopathologic study of 244 cases. *American Journal of Kidney Diseases*.

[22] Lehrer R. I., Jung G., Ruchala P., Andre S., Gabius H. J., Lu W. (2009). Multivalent binding of carbohydrates by the human *α*-defensin, HD5. *The Journal of Immunology*.

[23] Lehrer R. I. (2004). Primate defensins. *Nature Reviews Microbiology*.

[24] Leikina E., Delanoe-Ayari H., Melikov K. (2005). Carbohydrate-binding molecules inhibit viral fusion and entry by crosslinking membrane glycoproteins. *Nature Immunology*.

[25] Wang W., Owen S. M., Rudolph D. L. (2004). Activity of *α*- and *θ*-defensins against primary isolates of HIV-1. *The Journal of Immunology*.

[26] Moldoveanu Z., Wyatt R. J., Lee J. Y. (2007). Patients with IgA nephropathy have increased serum galactose-deficient IgA1 levels. *Kidney International*.

[27] Renfrow M. B., Cooper H. J., Tomana M. (2005). Determination of aberrant O-glycosylation in the IgA1 hinge region by electron capture dissociation fourier transform-ion cyclotron resonance mass spectrometry. *Journal of Biological Chemistry*.

[28] Takahashi K., Wall S. B., Suzuki H. (2010). Clustered O-glycans of IgA1: defining macro- and microheterogeneity by use of electron capture/transfer dissociation. *Molecular and Cellular Proteomics*.

[29] Gomes M. M., Suzuki H., Brooks M. T. (2010). Recognition of galactose-deficient *O*-glycans in the hinge region of IgA1 by *N*-acetylgalactosamine-specific snail lectins: a comparative binding study. *Biochemistry*.

[30] Moore J. S., Kulhavy R., Tomana M. (2007). Reactivities of N-acetylgalactosamine-specific lectins with human IgA1 proteins. *Molecular Immunology*.

[31] Mellis S. J., Baenziger J. U. (1983). Structures of the O-glycosidically linked oligosaccharides of human IgD. *Journal of Biological Chemistry*.

[32] Mattu T. S., Pleass R. J., Willis A. C. (1998). The glycosylation and structure of human serum IgA1, Fab, and Fc regions and the role of *N*-glycosylation on Fc*α* receptor interactions. *The Journal of Biological Chemistry*.

[33] Field M. C., Amatayakul-Chantler S., Rademacher T. W., Rudd P. M., Dwek R. A. (1994). Structural analysis of the N-glycans from human immunoglobulin A1: comparison of normal human serum immunoglobulin A1 with that isolated from patients with rheumatoid arthritis. *Biochemical Journal*.

[34] Arnett E., Seveau S. (2011). The multifaceted activities of mammalian defensins. *Current Pharmaceutical Design*.

